# American Medical Association, Section on Stomatology

**Published:** 1901-12

**Authors:** 


### AMERICAN MEDICAL ASSOCIATION, SECTION ON STOMATOLOGY.

### DISCUSSION ON PAPER ON "MILITARY DENTAL PRACTICE: ITS MODIFICATIONS AND LIMITATIONS," BY HENRY D. HATCH, NEW YORK CITY.

(For Dr. Hatch's paper, see page 749.)

*Dr. Eugene S. Talbot,* Chicago.—When this paper was received from Dr. Hatch it seemed a subject a little out of the general order, and hence that it would be wise to write to Dr. John S. Marshall, president of the Examining Board of Dental Surgeons, United States Army, and have him discuss the paper. His comments will now be read by Dr. A. H. Peck, of Chicago:

MR. CHAIRMAN AND MEMBERS OF THE SECTION ON STOMATOLOGY,—The passage of the Army Reorganization Bill with its section creating a corps of dental surgeons for the United States army makes an epoch in the history of modern dental surgery; an epoch which has never had its counterpart before in the history of the world, the influence of which is destined to be far reaching in its beneficent results, and of great importance in the elevation of our educational and professional standards.

When we take into consideration the fact that modern dentistry covers, in its growth and development, a period less than the lifetime of many individuals, we have good reasons to be proud of its achievements. Sixty years ago there was no such thing in existence as a dental college; a dental journal had not been thought of, nor a scientific dental society organized. The practice of dentistry at this time was, with few exceptions, in the hands of the barbers and blacksmiths. The few earnest, scholarly men who had entered its ranks were desirous of lifting their specialty out of its low estate, and by education and scientific training make it an honorable and worthy calling. In this endeavor, however, they met with no encouragement at the hands of their *confrères* in the medical profession, but, nevertheless, with these great odds against them, engendered not by malice, but by the narrow view taken by those unfamiliar with the capabilities and possibilities of development which lay in this infant specialty, they moved forward and laid the foundations of what has proved to be one of the greatest specialties in the whole field of medicine.

We have special reason for congratulation also in the fact that the Congress of these United States was the first legislative body in the world to formally recognize the *value* and *need* of the beneficent services of our specialty as a department of military medical practice, and that we have been given an opportunity to prove the wisdom of its action to our country and the world.

The whole question, however, of placing dental surgeons in the army was looked upon by many of our national legislators in somewhat of the light of an experiment, and in a certain measure this is true of the War Department and of the Surgeon-General. For this reason, when the bill was framed it was thought best to provide for the organization of the Dental Corps upon the contract system, as by this system it would be an easy matter to discontinue it if it should prove unsuccessful; while, upon the other hand, if its service was a success, and found to be an indispensable part of army medical practice, the Corps could be made permanent by amending the law so as to make its members commissioned officers.

The examinations of the Dental Corps and the selection of the outfits have been placed in the hands of an Examining and Supervising Board of Dental Surgeons, subject to the approval of the Surgeon-General and the Secretary of War. These officers have invariably sustained the board in all of its work; have given them every facility asked for, and rendered every assistance possible in planning the organization of the Corps and insuring its future success in the field.

European nations will be interested in the plan of organization of our Army Dental Corps, of its personnel, and in the results of its professional services. Upon the success or failure of this enterprise will depend in a large measure the introduction of a similar service into the armies of other nations. It is to be hoped, therefore, in the interests of humanity, that it may prove successful.

I have been interested in reading the paper of the essayist, and feel like complimenting him upon his apparent grasp of the subject under discussion. His suggestions as to the modifications and limitations of military dental practice are in many respects good, but it will not be necessary to confine army practice within such narrow limitations as he has outlined.

The general suggestions which he has made, and many more of a specific nature, have formed the subjects of much earnest consideration upon the part of Surgeon-General Sternberg and the Examining and Supervising Board, and not until each question has been carefully weighed and the consequences of its adoption or rejection examined from all stand-points has final action been taken. The progress of organization has been necessarily slow, for there were no established precedents to guide the board in its actions and no past experience to enlighten it. It has, therefore, been obliged to begin its work at the very foundation, striving to lay this *firm* and *sure,* and then gradually to rear upon this the superstructure of a finished organization. The organization of the Dental Corps is yet in the experimental stage; the authorities must, therefore, carefully feel their way and adapt the service to the peculiar demands of military life and movements, making such changes from time to time as experience and foresight shall dictate.

With the approval of the Surgeon-General, I herewith present in brief outline the plan upon which the Army Dental Corps is organized. This outline comprises:

1. The official status and pay of the army dental surgeon.
2. The examination of candidates.
3. Assignment to duty.
4. Regulations governing the Dental Corps.
5. The supply table.
6. Nosological table and system of keeping records.

### THE OFFICIAL STATUS.

The army dental surgeon belongs to the regular establishment, and, according to the law which created the Corps, he will serve the officers and enlisted men of the regular and volunteer armies. He is employed as a contract dental surgeon, having the *relative* rank of a first lieutenant. His pay is one hundred and fifty dollars ($150) per month, and quarters when serving with troops. All instruments, apparatus, and materials that are necessary for conducting his practice and performing his official duties are furnished by the Medical Department.

### EXAMINATION OF CANDIDATES.

The examinations consist of (*a*) physical condition; (*b*) written and oral questions upon the studies of the dental college course;

(*c*) practical demonstrations in operative dentistry; (*d*) practical demonstrations in prosthetic dentistry.

(*a*) The physical examination is conducted by an army surgeon detailed for this purpose, and upon the same general lines as those in vogue for entrance into the other departments of the army. Perfect health and freedom from physical defect are necessary to pass this examination; but defective eyesight which can be corrected by appropriate glasses does not debar the candidate.

(*b*) Written and oral examinations are conducted upon the following-named subjects, and the candidate must attain a general average of seventy-five per cent. upon each of them: Anatomy, physiology, histology, chemistry, physics, metallurgy, dental anatomy and physiology, dental materia medica and therapeutics, dental pathology and bacteriology, orthodontia, oral surgery, operative dentistry, prosthetic dentistry.

(*c*) The practical examination in operative dentistry consists of: 1. Examination and recording the condition of the mouth and teeth. 2. Preparation of cavities (*a*) by hand instruments; (*b*) by engine instruments. 3. Instrumentation and technique. 4. Preparation and manipulation of filling-materials: gold, tin, amalgam, gutta-percha, oxyphosphate, cement. 5. Inserting and finishing of fillings. 6. Treatment and filling of root-canals and preparation of root for pivot crown. 7. Manipulative technique in removal of calcareous deposits. 8. Application of rubber dam, metallic separators, matrices, etc. 9. Diagnosis, prognosis, and treatment of oral diseases. 10. Care of and sterilization of instruments and hands.

(*d*) The practical examination in prosthetic dentistry comprises: 1. Impressions, casts, bite and articulation (occlusion). 2. Construction of denture in vulcanite. 3. Construction of dies and counter-dies from impression to completion. 4. Construction of swaged plate, with metal and rubber attachment. 5. Construction of interdental splints. 6. Construction of Richmond crown.

Upon all clinical or practical demonstrations the candidate must attain a general average of eighty-five per cent. The reason for this higher requirement in the practical branches is made evident by the statement that practical men are needed in the service, not theorists. But it may also be stated that practical ability alone would be of little value in military dental practice. The dentist to be successful in this new field of practice must be thoroughly informed upon all those subjects and theories which form the foundation of modern dental surgery. He will need to be self-reliant and capable of conducting any case that may come under his especial care, no matter how serious it may be, for he will many times be so located that he cannot obtain the advice of a consultant in his specialty; while, upon the other hand, he may be, and that frequently, called in by the post surgeon as a consultant in cases which present oral or dental lesions; or which by reason of certain symptoms the surgeon is led to believe may be dependent upon some obscure dental or oral malady and upon which he desires an expert opinion to assist him in an intelligent treatment of the case.

ASSIGNMENT TO DUTY.

As rapidly as the candidates have passed the examining board they have been assigned to service in the Philippines and Cuba, for the reason that our troops located in tropical climates are in the greatest need of the services of the dentist. When these demands are satisfied in one location the dentist will be assigned to another station, and thus moved about from place to place, wherever his services are most urgently needed. The number of dentists is so few, thirty in all, and the army so large, something over seventy thousand, that there is likely to be an abundance of employment for all those who enter this service. The opportunity, however, for a large and varied scientific and practical experience along professional lines, offered by the establishment of the Army Dental Corps, will be very great, and ought to prove a tempting inducement to the really progressive young man.

REGULATIONS GOVERNING THE DENTAL CORPS.

The following regulations having received the approval of the Honorable the Secretary of War, become, with the General Regulations bearing upon officers of the army, the law governing contract dental surgeons:

Candidates for appointment as dental surgeons must not be less than twenty-four nor more than forty years of age. They must be graduates of standard medical or dental colleges, trained in the several branches of dentistry, of good moral and professional character and prior to appointment will be required to pass a satisfactory professional examination before a board of dental surgeons convened for that purpose by the Secretary of War.

Contracts with dental surgeons will be made for three years, but may be annulled at any time by the commanding general of a military department, after official investigation, for conduct to the prejudice of good order and military discipline, or by the Surgeon-General when in his opinion a termination of the contract would be in the interests of the service.

Dental surgeons are attached to the medical department and will be assigned to duty in accordance with the recommendations of the Surgeon-General of the army or the chief surgeon of a military department.

A dental surgeon when assigned to a station will apply to the post commander for a suitable operating-room. If no other room is available, the surgeon of the post may assign him a room in the hospital.

Each dental surgeon will ordinarily be allowed one enlisted man as assistant, who will be detailed from the acting hospital stewards or privates of the Hospital Corps, and whose duty it will be to assist the dentist in his operations, in caring for the instruments and other public property, in keeping the records, and in the performance of such other official work pertaining to this position as he may be directed by the proper authority to do. When a member of the Hospital Corps is detailed as dentist's assistant he will receive commutation of rations at the rate of one dollar daily, and will be provided with a suitable room as quarters by the Quartermaster's Department, except while on duty at a post, when he will be attached to the Hospital Corps or other organization for rations and quarters.

Necessary dental instruments and supplies will be purchased by medical supply officers under instructions from the Surgeon-General and in accordance with a supply table to be approved by the Secretary of War.

Dental surgeons will be held strictly responsible for all instruments and supplies issued to them, and will be governed by army regulations and orders now in force, or hereafter to be issued, with reference to accountability for government property.

In accordance with the act of Congress authorizing their employment, dental surgeons will " serve the officers and enlisted men of the regular and volunteer army." The families of officers and civilian employees attached to the army are not entitled to their services. In this connection acting assistant surgeons are to be regarded as commissioned officers.

Dental surgeons will operate between the hours of nine A.M. and four P.M. only upon those officers and enlisted men who are entitled to their services. They may operate upon others not entitled to free services before and after these hours when their services are not required by those entitled to them, but material issued to them by the government will only be used in operations upon officers and enlisted men of the army.

Dental surgeons will not perform any operation upon officers or enlisted men of the army or prescribe medicines for them other than those necessary for the treatment of the teeth and gums. This prohibition does not apply to cases of emergency where no medical officer is within reach, and where a dental surgeon is able to render the necessary surgical assistance to meet the immediate emergency.

Emergency work, whether for officers or enlisted men, should always have precedence. Plate work or restoration of teeth by any method will only be done for those who have lost teeth in the service and in the line of duty. For plate work or filling teeth only the cheaper materials will be supplied, but gold may be used, if the operating dentist sees fit to use it, at the expense of the individual operated upon.

Enlisted men requiring the services of the dental surgeon will, at the hour prescribed by the commanding officer, be conducted to the designated place under a non-commissioned officer, who will take with him and hand to the dentist a list of those reporting for treatment. This list will be entered in a daybook ruled in columns for surname, given name, rank, company, regiment, etc.; all headings to be the same as those borne on his monthly report.

All cases requiring treatment involving future appointment will be so noted, and the others will be marked according to the circumstances, as "treatment unnecessary," "further treatment unnecessary," "should be sent to the surgeon," etc. When future treatment is necessary the dentist will forward a card as follows:

............................19....

The Adjutant:

...............................................

Sir,—I have the honor to ask that..............................
be directed to report to me from........m. to........m. on..............
instant for treatment.

Very respectfully,

..............................

*Dental Surgeon.*

Dental surgeons will submit a monthly report in duplicate (on prescribed blanks) of all official work done by them, giving all required data in every case in which professional services have been rendered. This report will be an exact copy of the register kept for the period. One copy will be sent on the last day of the month to the Surgeon-General and one to the chief surgeon of the department in which the dental surgeon is serving.

### THE SUPPLY TABLE.

This table is too long to present in this place, but it may be stated that it includes a portable dental chair and a dental engine, packed in separate cases; burs, mandrels, stones, disks, etc.; excavators, chisels, scales, plastic-pluggers, gold-pluggers, rubber-dam clamps, clamp forceps, dam punch, extracting forceps, elevators, steam sterilizer, etc.; in fact, all of the instruments and adjuncts that are really necessary to perform any operation upon the teeth, except for crowns and bridges. Each outfit contains medicines and supplies of filling-material sufficient for three months' service. The smaller instruments and the supplies are packed in two strong cases, arranged with trays and receptacles to hold the instruments and the supplies in place. The whole outfit when cased and crated weighs about four hundred and fifty pounds. To protect the cases from rain and dampness they are enclosed in canvas covers. The general hospitals, and such other posts as may be designated by the Surgeon-General will be furnished with an additional outfit, consisting of a regular office operating-chair, Allan bracket, cuspidor, instrument-case, extra extracting forceps, and a full laboratory outfit for constructing vulcanite plates, swaged metal plates, interdental splints, crowns, and bridge-work.

### NOSOLOGICAL TABLE AND SYSTEM OF KEEPING RECORDS.

The ordinary system of keeping records in civil practice, by means of charts, could not be employed in military practice, for the reason that it would occupy too much space. As a substitute for this the following system has been devised and has received the approval of the Surgeon-General:

#### DISEASES AND INJURIES OF THE TEETH AND MOUTH.

Abrasion (mechanical).
Abscess of the jaws (associated with impacted teeth).
Calcification of the pulp.
Caries.
Cysts of the jaws (associated with devitalized teeth).
Dento-alveolar abscess.
Erosion (chemical).
Fractures of the teeth.
Hemorrhage (following extraction).
Hypertrophy of the pulp.
Hypertrophy of the gums.
Hypercementosis.
Necrosis of the teeth.
Pericementitis, acute.
Pericementitis, chronic.
Pulpitis, acute.
Pulpitis, chronic.
Pyorrhœa alveolaris.
Resorption of the alveolar processes.
Salivary deposits.

NOTE.—The duties of the dental surgeon will ordinarily be confined to the treatment of such cases as are directly associated with the teeth and gums; but occasions may arise when his services would be required as a specialist in the treatment of diseases and injuries of the mouth and jaws, such as cysts of the salivary ducts, empyema of the maxillary sinus, fractures of the jaws, gingivitis, necrosis of the jaws, facial neuralgia, stomatitis, and tumors of the gums and jaws, etc.

CLASSIFICATION OF THE TEETH.

| | |
|---|---|
| 1. Superior central incisors. | 9. Inferior central incisors. |
| 2. Superior lateral incisors. | 10. Inferior lateral incissors. |
| 3. Superior cuspids. | 11. Inferior cuspids. |
| 4. Superior first bicuspids. | 12. Inferior first bicuspids. |
| 5. Superior second bicuspids. | 13. Inferior second bicuspids. |
| 6. Superior first molars. | 14. Inferior first molars. |
| 7. Superior second molars. | 15. Inferior second molars. |
| 8. Superior third molars. | 16. Inferior third molars. |

In designating the teeth and in recording all operations upon them, the dental surgeon will indicate the tooth by the following plan, using the letters R. and L. to designate the right and left sides and the figures 1, 2, 3, etc., to designate the tooth. Examples: R. 1, Right superior central incisor; L. 14, Left inferior first molar.

CLASSIFICATION OF CAVITIES.

*Simple Cavities on Exposed Surfaces.*

| Incisors and Cuspids. | Bicuspids and Molars. |
|---|---|
| A. Labial. | D. Morsal. |
| B. Lingual. | E. Buccal. |
| C. Morsal. | F. Lingual. |

*Simple Approximate Cavities.*

| Incisors and Cuspids. | Bicuspids and Molars. |
|---|---|
| G. Mesial. | I. Mesial. |
| H. Distal. | J. Distal. |

*Compound Cavities.*

| Incisors and Cuspids. | Bicuspids and Molars. |
|---|---|
| K. Mesio-labial. | R. Mesio-morsal. |
| L. Disto-labial. | S. Disto-morsal. |
| M. Mesio-lingual. | T. Morso-buccal. |
| N. Disto-lingual. | U. Morso-lingual. |
| O. Mesio-morsal. | V. Mesio-disto-morsal. |
| P. Disto-morsal. | W. Bucco-linguo-morsal. |
| Q. Mesio-disto-morsal. | |

In recording all operations of filling the teeth, the cavity will be described by the dental surgeon according to the preceding classification, using the letters A, B, C, etc., to designate its location.

*Example:* A, Simple cavity in labial surface of an incisor or cuspid tooth. I, Simple cavity in mesial surface of a bicuspid or a molar. V, Compound cavity in mesial, distal, and morsal surfaces of a bicuspid or a molar.

CLASSIFICATION OF FILLING MATERIAL.

Tin.
Amalgam.
Oxyphosphate.
Gutta-percha.

The kind of filling-material employed will be indicated by using the first letter of the word designating that material.

*Example:* R. 5 Q. A. Tooth, right, superior second bicuspid; cavity, mesio-disto-morsal surfaces. Filling-material, amalgam. If a combination filling is employed it will be designated by the first letters of the words designating the materials used. *Example:* L. 7 S. G.-O. Tooth, left superior second molar; cavity, disto-morsal surfaces. Filling-material, gutta-percha and oxyphosphate cement.

*Exception:* The only filling-materials furnished for the use of the dental surgeon by the Medical Department are those enumerated above. Gold, however, may be provided by the dental surgeon and inserted for those officers and enlisted men who are willing to reimburse him for its cost. The minimum fee to be one dollar, and the maximum two dollars, for such filling.

In recording operations made with gold the full word should be written out.

Other operations upon the teeth will be designated by a combination of letters, as follows:

Abscess lanced—A-L.
Calculus removed—C-R.
Pulp devitalized—P-D.
Pulp extirpated—P-E.
Root-canal filled—R-F.
Tooth extracted—T-E.
Tooth treated—T-T.

One hundred of these sheets are bound in a book, and form the Register of Dental Operations. Each month the dental surgeon is required to send to the Surgeon-General an exact transcript of the Register of the operations performed during that month, upon blank sheets known as the Monthly Record of Dental Operations. These reports are placed on file for future reference. The Register when filled is transmitted to the Surgeon-General and also placed on file.

These records will form an important means of identification in cases of death or desertion, while to the Pension Office they will prove of value in passing upon those applications for pension which are based upon the loss of teeth while in the service of the United States army.

They will also prove of immense value from the scientific standpoint, in efforts to settle the questions of the relative prevalence and spread of dental caries among men who have been selected for military service, because of their perfect physical condition, as compared with men in civil life; of the relative increase in this disease resulting from physical and nervous strain of severe campaigns, and of residence in tropical climates; of the causes and prevalence of pyorrhœa alveolaris, gingivitis, stomatitis, and kindred oral affections.

It will be noticed by the Regulations governing the Army Dental Surgeons and the rules laid down by the Surgeon-General, that the only filling-materials which will be furnished by the government are tin and the plastics; but the dentist is not prohibited from using gold, if the officer or the enlisted man is willing to reimburse him for the material used. (This material he must carry with him at his own risk.) In fact, he is encouraged to use gold by being furnished with instruments and applianes with which to perform this class of operations. He is, furthermore, furnished with an acting hospital steward, whom he is expected to train as an assistant. With such intelligent assistance as this, there is no reason why as good gold fillings may not be inserted by the military dental surgeon as by the dentist in civil practice.

The character of the service which must be rendered in the field will of necessity be that of meeting emergencies. Relief from suffering will be the first object of the treatment, and the introduction of temporary fillings to protect the teeth from further decay until a more favorable opportunity can be secured for inserting a permanent filling.

The dental surgeon will, however, as a rule, be located at posts and stations of a more or less permanent character, where he can conduct his practice with as much care and thoroughness as is possible with the civil dental surgeon.

Each field outfit is provided with a quantity of modelling composition and impression-trays, so that fractures of the maxilla can be treated temporarily, by interdental supports made from the modelling composition, while the patient is being transported to the general hospital, where facilities will be found for constructing any form of interdental splint or other mechanical apparatus that might be indicated.

The army dental surgeon is expected to confine his professional services to the treatment of the diseases of the teeth and their associated parts, but the regulations provide that in cases of emergency, when no surgeon is present, he may, if competent, render any assistance that the case may demand.

The dental surgeons located at general hospitals and large posts will be furnished with as good and as complete an outfit for a general dental practice as is found in the offices of the best civil practitioners. At such stations the dental surgeons will have opportunities for the treatment of cases in Orthodontia for the children of the officers of the station, or inserting crowns, bridges, and artificial dentures for the officers and enlisted men and for their families; while the general hospitals will furnish a certain number of cases of fractured maxilla, that will need to be treated by the construction and insertion of some one of the various forms of interdental splints.

From the foregoing pages it will be seen that the War Department, through the recommendations of the Surgeon-General, has provided for the care and the treatment of the dental and oral diseases of the army in as thorough and as scientific a manner as is possible under the exigencies of military life and movements. Experience, however, may make it necessary to institute certain changes and modifications in the present system of service, and, when such action is proved to be essential to the welfare of the army and an increased efficiency of the Corps, there is no doubt that the proper authorities will immediately institute such changes and modifications.

*Dr. Eugene S. Talbot,* Chicago.—It occurred to me after that bill was passed that the profession knew very little about the work to be done by the dentist appointed to fill the position in America's new possessions. It is very gratifying to know that Dr. Marshall has received that appointment. The fact that so few men have been appointed or accepted who have applied augurs well for the standard the board has adopted. They have already stirred up quite a little commotion in dental journals. Editorials have been written and letters have been received showing there is uneasiness. The average dental teacher is of the opinion that all there is of dentistry is simply the filling and extraction of teeth. It is very fortunate for the education of the profession that this law has been passed and a number of dentists sent to the field to attend to soldiers. The work of filling teeth is a very small part of the work these dentists are called upon to perform. It has been shown, not only in Cuba, but in the Philippines, that many diseases, especially gingivitis, affect the jaws of people who suddenly change climate. It is due to the way the excretory organs are affected by the sudden change of climate; the jaws and the teeth become involved, and in a very short time, sometimes in the course of a few weeks, inflammation sets in, teeth become loose, and in many cases drop out. Such conditions appear in the report that Dr. Marshall has made. The fact that a dentist is expected not only to fill teeth and make plates, but to perform other duties ordinarily within the scope of a physician or surgeon, demands that dentists be properly educated along these lines. The average physician has to be posted in the treatment of those apparently new diseases. A man to treat these cases intelligently must understand general medicine. Just how far a dentist would be allowed the conduct of these cases and give treatment remains to be seen. The conditions shown by Dr. Marshall makes it evident that dental colleges will have to advance their course in pathology, and that a four years' course is now necessary.

